# HIV incidence among men who have sex with men using geosocial networking smartphone application in Beijing, China: an open cohort study

**DOI:** 10.1186/s40249-021-00814-7

**Published:** 2021-04-02

**Authors:** Guo-Dong Mi, Bin-Bin Zhang, Fei Yu, Xian-Long Ren, Jason J. Ong, Ya-Qi Fan, Feng-Hua Guo, Chun-Jun Li, Mian-Zhi Zhang, Min-Ying Zhang

**Affiliations:** 1 Danlan Beijing Media Limited, Beijing, China; 2 School of Medicine, Nankai University, 94, Weijin Road, Tianjin, 300071 China; 3 Beijing Center for Diseases Control and Prevention, Beijing, China; 4 Central Clinical School, Monash University, Melbourne, Australia; 5 Department of Clinical Research, London School of Hygiene and Tropical Medicine, London, UK; 6 Tianjin People’s Hospital, Tianjin, China; 7 Dongfang Hospital, Beijing University of Chinese Medicine, Beijing, China

**Keywords:** Men who have sex with men, Geosocial networking application, HIV, Incidence, Sexual risk behavior

## Abstract

**Background:**

Sexual transmission among men who have sex with men (MSM) is the dominant route of HIV transmission in China. Extensive use of geosocial networking (GSN) smartphone application (app) has dramatically changed the pattern of sexual behaviors and HIV risk among MSM, but data on HIV incidence and the changing risk behaviors of GSN app-using MSM are limited. We aims to assess the HIV incidence and its correlates among gay GSN app-using MSM in China.

**Methods:**

We constructed an open cohort which was initiated and maintained using a GSN app to assess the HIV incidence among app-using MSM, recruited from June 2017 to December 2018. MSM completed an online questionnaire on their sociodemographic characteristics, sexual behaviors, recreational drug use and sexually transmitted infections status. Then each man had an HIV test, and those tested negatives were enrolled into the cohort. Participants completed follow-ups with additional HIV tests though the app during the study period, and were censored at HIV seroconversion or study end date. HIV incidence was calculated by dividing the sum of observed HIV seroconversions by the observed person-time. Univariate (Chi-square test and Fisher’s exact test) and multivariate (proportional hazards regression) analyses were used to examine correlates of HIV incidence.

**Results:**

A total of 6957 HIV negative MSM were enrolled in the open cohort, 37 seroconversions occurred among 1937 men contributing 1065 observed person-years: HIV incidence was 3.47 per 100 person-years [95% confidence interval (*CI*): 2.37–4.57]. More than five sexual partners [hazard ratio (HR) = 2.65, 95% *CI*: 1.04–6.67], and sex with HIV positive partners (HR = 3.82, 95% *CI*: 1.16–12.64) in the preceding six months were positively associated with HIV seroconversion. Consistent condom use for anal sex (HR = 0.27, 95% *CI*: 0.07–0.96), and reporting insertive anal sex only (HR = 0.23, 95% *CI*: 0.08–0.62) in the preceding six months were protective factors for HIV seroconversion.

**Conclusions:**

Tailored interventions targeting app-using MSM are urgently needed given their high risk of HIV. As a new tool for accessing MSM at higher HIV risk, GSN smartphone app could play an important role in HIV research among MSM. 
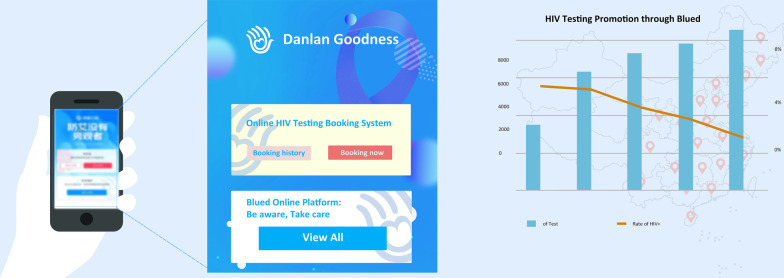

## Background

Men who have sex with men (MSM) are a key population for HIV prevention and control. The Global AIDS report released by the Joint United Nations Programme on HIV/AIDS (UNAIDS) estimated that MSM have a 26 times higher risk of acquiring HIV than general population [1]. Sexual transmission among MSM is the dominant route of HIV transmission in China [2]. Although there are robust data on HIV prevalence among MSM in China [2, 3], data for incidence is scarce. Understanding HIV incidence (particularly among subgroups of key populations) is important for efficient targeting of strategies to prevent new HIV infections. A prospective cohort study is recommended to estimate HIV incidence, but it is challenging to maintain cohorts for MSM in China due to their fear of stigma and discrimination and perceived lack of confidentiality. Most reports about HIV in China come from cross-sectional studies. The MSM cohort studies published in China so far might not be completely reliable due to their small sample sizes (*n* = 155–547), short follow-up period (mostly 3–6 months) and high loss of follow-up rate (28.5–56.6%) [4–9].

The limited data from cohort studies show that HIV incidence varies widely. In 2014, one cohort observed HIV incidence among MSM to be as high as 13.6/100 person-years in a city of Jiangsu province [10]. A study with data from eight Chinese cities from 2016 to 2017 reported an incidence of 15.6/100 person-years [11]. HIV incidence has geographical variations among different regions in China [12]. Higher HIV incidence is reported in metropolitan cities compared to smaller cities [3]. Therefore, Beijing, the capital of China—experiencing a large influx of MSM every year—is expected to have the highest HIV incidence, but this has not corresponded with the current literature. For example, a cohort study with 525 MSM in Beijing during 2008–2009 observed an HIV incidence of 3.4/100 person-years with 457 MSM completing the 12 month follow-up [13]. A cohort with 1003 MSM recruited in Beijing (2009–2012) observed an HIV incidence of 6.0/100 person-years [95% confidence interval (*CI*): 4.2–8.4] with only 699 participants completing the follow-up (loss-to-follow-up rate: 30.3%) [14]. However, given the small number of participants and the observed person years, the results of these cohort studies may be inaccurate and unreliable.

It is challenging to construct mutual trust between clinical professionals, public health practitioners and MSM due to their fear of stigma and discrimination, which also makes it difficult to maintain a MSM cohort. Therefore, new strategies to recruit and maintain a cohort of Chinese MSM are needed. MSM were the early adopters of the internet [15], and the internet has become primary sources of social support for MSM [15, 16]. Internet-based recruitment can be more efficient and cost-effective in terms of recruitment than field-based recruitment among population with high-risk behaviors [16, 17], opening the possibility for constructing an MSM cohort.

Constructing an online cohort of Chinese MSM also has the advantage of reaching a relatively high-risk group of MSM. With the popularity of smartphones—manifested by the proliferation of GSN smartphone apps, such as Blued, Grindr and Jack’d—increasing use of these apps among MSM has facilitated the ease of finding casual sexual partners. Previous studies have reported that MSM who used apps had more sexual encounters, more frequent anal intercourse, more condomless anal intercourse, and a larger number of sexual partners living with HIV [18–23]; thus increasing their risk for HIV and sexually transmitted infections (STIs), compared with MSM who did not use apps to seek sex partners[19, 24–27]. However, results about sexual behaviors and HIV/STIs prevalence among app-using MSM are inconsistent. Some studies suggested that app-using MSM were more likely to use condoms with their partners than non-app users [28, 29], have better HIV testing behaviors [25], and that use of apps was not associated with increased risk for HIV or STIs [30–32]. The patterns of sexual partner seeking behaviors among MSM have changed dramatically from venue-based to internet-based, especially GSN smartphone app-based, which could facilitate sex partner seeking and result in casual sex. But the influence of risky sexual behaviors on HIV incidence caused by this change has not been clearly established, and the current data on HIV incidence among GSN smartphone app-using MSM in China are insufficient to understand the current HIV epidemic and risk sexual behaviors. The only paper we are aware of that reported HIV incidence among GSN app-using MSM found that new HIV infections were independently associated with ever using GSN apps, but their sample size was small (276 app-using MSM and 185 nonusers) and app using behavior was self-reported [33]. So, reliable data on the HIV incidence and the changing sexual behaviors of GSN app-using MSM are urgently needed.

To address these gaps, our study aims to: (1) construct the first gay GSN app-using MSM cohort in China; (2) accurately characterize HIV incidence and its correlates among gay GSN app-using MSM; and (3) summarize the characteristics of GSN app-using MSM.

## Methods

### Study site

We conducted an open cohort study in Beijing, China. As the political, economic and cultural center of China, Beijing has a population of 21.54 million people, among which 7.65 million are internal immigrants [34]. Beijing was purposively selected for it has a large influx of MSM every year [35–38].

### Study design

We constructed an open cohort of MSM since June 2017; inviting visitors to Blued, a gay GSN app with 40 million users worldwide, to participate in the cohort. After completing an online self-administrated questionnaire via the app which collected data on sociodemographic characteristics, sexual behaviors, recreational drug use and STIs status, participants could then make an appointment for an HIV test in one of the four HIV screening sites set up by Blued in Beijing. HIV screening was conducted by trained peer testers from Blued with finger-prick blood sample. We used a rapid HIV test (Colloidal Gold Device) manufactured by Beijing Wantai Biological Pharmacy Enterprise Co., Ltd, which was accepted for the World Health Organize list of prequalified in vitro diagnostics. MSM with positive screening results were referred to the appropriate district center for disease control (CDC) in Beijing for HIV confirmatory test using Western blot test.

A baseline survey was completed when a participant finished the online survey and received an offline HIV test through Blued for the first time during the study period; MSM with questionnaires completed within thirty seconds or incomplete questionnaires were considered likely to be untruthful or not serious and were excluded. Subsequently, additional HIV testing together with the completion of the online survey by the same study subject was regarded as the completion of a follow-up visit. We report data up to December 31, 2018 and participants were censored at HIV seroconversion or December 31, 2018, whichever came first. The follow-up time of those who received more than two HIV tests by Blued during the study period was calculated as the time interval between the latest HIV test and the baseline survey. MSM included in the cohort who tested positive for HIV during follow-up were counted as one positive seroconversion of HIV. Our study protocol was approved by Nankai University Health Research Ethics Committee and electronic individual informed consent was obtained before the online survey.

To improve the data quality, no incentives offered for participation in our survey. But participants received private messages, messages on the startup screen, advertisement banners, and invitations to live streaming broadcasts through Blued where MSM were encouraged to test for HIV to reduce the loss to follow-up.

### Participant eligibility criteria

Men who met the following criteria were recruited into our cohort: (1) born biologically male; (2) aged ≥ 18 years; (3) ever had sex with men; (4) resided in Beijing with a Blued account registered in Beijing; (5) finished the online survey and offline HIV test during the study period; and (6) voluntarily participated in this study and signed the online informed consent. MSM were excluded if they: (1) tested HIV positive at baseline; (2) completed questionnaires within 30 s or had incomplete questionnaires.

### Outcome variables

The primary outcome was HIV seroconversion. We also collected information on their sexual behaviors in the preceding six months including having anal sex intercourse (yes, no), number of sex partners (one, two to five, six or more), knowledge of their sex partners’ HIV status (yes, no), having sex partners who were living with HIV (yes, no, not sure), frequency of condom use during anal sex (never, sometimes, every time), role during anal sex (exclusively receptive, exclusively insertive, versatile), heterosexual sex (yes, no), frequency of condom use during heterosexual sex (never, sometimes, every time), participation in group sex (yes, no), diagnosed with other STIs (yes, no) and recreational drug use (yes, no). We also collected participants’ sociodemographic characteristics, including age (years), highest level of education (high school or below, college/undergraduate, postgraduate), current employment status (worker, service sector, civil servant, student, company employee, freelancer, other), and duration of residence in Beijing (less than six months, 6–11, 12–23, 24 months or more, and local resident).

### Statistical analysis

Individual observation time was calculated as the interval between the participant’s baseline survey and the latest HIV test during the study period. HIV incidence was calculated by dividing the sum of observed HIV seroconversions by the observed person-time. The Kaplan–Meier cumulative probability of HIV seroconversion was presented using a survival curve. We used descriptive statistics to summarize the participants’ sociodemographic characteristics, sexual behaviors, recreational drug use and past diagnosis of STIs in the last six months before their latest HIV test. Frequencies and percentages were used to describe categorical variables. Median and interquartile range (IQR) was presented for age, as it was non-normally distributed. Quantitative variables like age, duration of residence in Beijing, number of sex partners during the last six months before the interview were converted to categorical variables. Sociodemographic characteristics, sexual behaviors, recreational drug use and being diagnosed with STIs were used as independent variables, while HIV seroconversions was used as the dependent variable to examine correlates of HIV incidence using Chi-square test and Fisher’s exact test. Statistically significant independent variables identified from the univariate analysis with *P* < 0.10 were included in a proportional hazards regression model for multivariate analysis to identify the correlates of HIV seroconversion; the final model contained covariates with *P* < 0.05. Statistical analysis was performed using Stata (Version 12, College Station, TX, USA).

## Results

### The cohort and HIV seroconversion

**Fig. 1:**
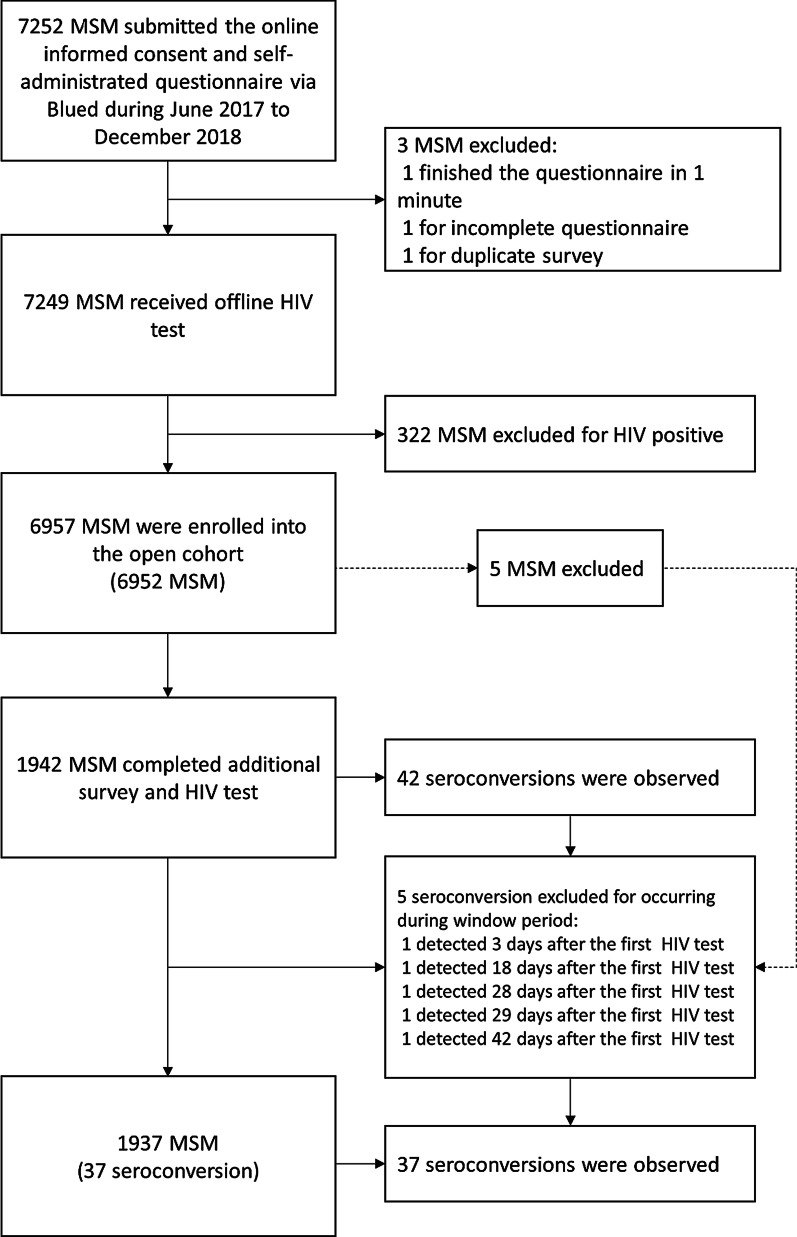
Flowchart of study population selection for GSN app using men who have sex with men (MSM) in Beijing, China

**Fig. 2:**
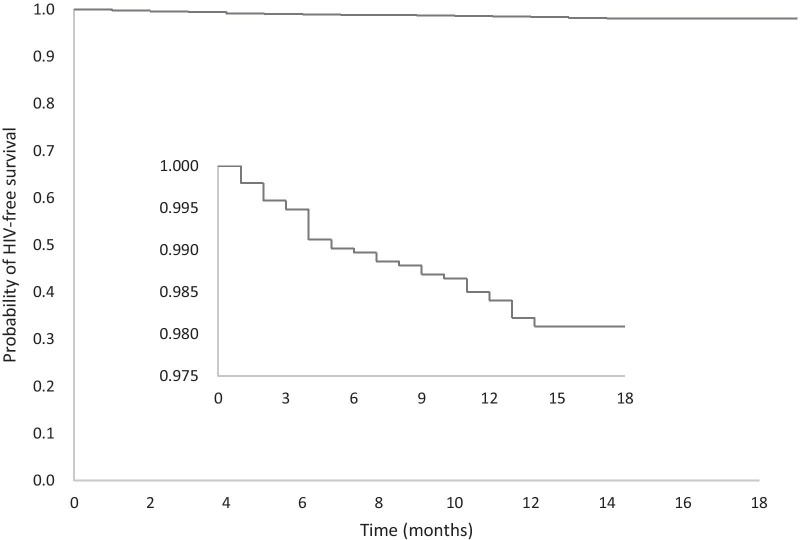
HIV incidence among geosocial networking smartphone application using men who have sex with men in China, probability of HIV-free survival

In total, 7252 MSM submitted the online informed consent and questionnaire. After excluding one man for an incomplete survey, one for completing the survey within thirty seconds, one for duplicated survey, 7249 MSM received offline HIV test. After excluding 322 who tested HIV positive, we enrolled 6957 HIV negative men in the cohort. After excluding another five seroconversions which occurred within the window period (≤ 45 days), our cohort identified 37 HIV seroconversions among 1937 HIV negative MSM who reported two or more episodes of HIV testing during the study period. Figure 1 shows the selection of our study population. The follow-up encounters ranged from 1 to 10, while the follow up interval ranged from 1 to 18 months with a median of 5.97 months (IQR: 3.00–10.00). The total person-time observed was 1065 person-years. The HIV incidence rate was 3.47 per 100 person-years (95% *CI*: 2.37–4.57). Figure 2 shows the Kaplan–Meier cumulative probability of HIV-free survival.

### Demographic characteristics of the cohort members (Table 1)

**Table 1:** Demographic characteristics of the participants and the results of the univariate analysis of demographic characteristics and HIV seroconversion.

	All *n *(%)	HIV seroconversion		*P* value
		Yes	No	
		*n* (%)	*n* (%)	
Age (years)				0.91
18–26	954 (49.3)	17 (46.0)	937 (49.3)	
27–36	775 (40.0)	16 (43.2)	759 (40.0)	
37–60	208 (10.7)	4 (10.8)	204 (10.7)	
Education level				0.15
High school or below	253 (13.1)	7 (18.9)	246 (13.0)	
College & undergraduate	1304 (67.3)	27 (73.0)	1277 (67.2)	
Postgraduate	380 (19.6)	3 (8.1)	377 (19.8)	
Duration of residence in Beijing				0.47
< 6 months	169 (8.7)	5 (13.5)	164 (8.6)	
6–11 months	132 (6.8)	1 (2.7)	131 (6.9)	
12–23 months	877 (45.3)	17 (46.0)	860 (45.3)	
24 months or more	590 (30.5)	9 (24.3)	581 (30.6)	
Local resident	169 (8.7)	5 (13.5)	164 (8.6)	
Occupation*				0.07
Workers	44 (2.3)	1 (2.7)	43 (2.3)	
Service sector	105 (5.4)	2 (5.4)	103 (5.4)	
Civil servants	154 (8.0)	1 (2.7)	153 (8.1)	
Students	186 (9.6)	3 (8.1)	183 (9.6)	
Company employees	471 (24.3)	3 (8.1)	468 (24.6)	
Freelancers	116 (6.0)	4 (10.8)	112 (5.9)	
Other	861 (44.4)	23 (62.2)	838 (44.1)	

*Hypothesis testing of rate/proportion differences using Fisher’s exact test

The median age of cohort participants was 26.68 (IQR: 23.47–31.02) years. Participants who were 18–36 years old accounted for 87.7%. The majority (86.9%) of participants completed college or university education. Most (91.3%) participants immigrated from another province, and 60.8% lived in Beijing for less than two years.

### Sexual behaviors, recreational drug use and being diagnosed with STIs in the last 6 months before the latest HIV test (Table 2)

**Table 2:** Sexual behaviors, STIs and recreational drug use of the participants in the last six months and univariate analysis of sexual behaviors and HIV seroconversion.

	All *n* (%)	HIV seroconversion		*χ*^*2*^	*P* value
		Yes *n* (%)	No *n* (%)		
Number of sexual partners				8.72	0.01
0–1	1217 (62.8)	17 (45.9)	1200 (63.2)		
2–5	603 (31.1)	14 (37.9)	589 (31.0)		
≥ 6	117 (6.0)	6 (16.2)	111 (5.8)		
HIV-positive partners				8.12	0.02
None	915 (47.2)	10 (27.0)	905 (47.6)		
Yes	87 (4.5)	4 (10.8)	83 (4.4)		
Not sure	935 (48.3)	23 (62.2)	912 (48.0)		
Anal sex				1.37	0.24
Yes	1869 (96. 5)	37 (100.0)	1832 (96.4)		
No	68 (3.5)	0 (0.0)	68 (3.6)		
Using condom for anal sex				11.79	<0.01
Never	68 (3.6)	3 (8.1)	65 (3.6)		
Sometimes	601 (32.2)	20 (54.1)	581 (31.7)		
Every time	1200 (64.2)	14 (37.8)	1186 (64.7)		
Sex role for anal sex				9.55	0.01
Exclusively receptive	215 (13.1)	15 (40.5)	497 (26.2)		
Exclusively insertive	723 (44.1)	5 (13.5)	718 (37.8)		
Versatile	702 (42.8)	17 (46.0)	685 (36.0)		
Group sex				0.34	0.56
Yes	159 (8.2)	4 (10.8)	155 (6.9)		
No	1778 (91.8)	33 (89.2)	1745 (93.1)		
Heterosexual sex				0.35	0.55
Yes	638 (51.6)	10 (58.8)	628 (51.6)		
No	597 (48.4)	7 (41.2)	590 (48.4)		
Using condom for heterosexual sex				9.63	0.01
Never	83 (13.0)	4 (40.0)	79 (12.6)		
Sometimes	156 (24.5)	4 (40.0)	152 (24.2)		
Every time	399 (62.5)	2 (20.0)	397 (63.2)		
Diagnosed with a sexually transmitted infection				4.88	0.03
Yes	75 (3.9)	4 (10.8)	71 (3.7)		
No	1862 (96.1)	33 (89.2)	1829 (96.3)		
Recreational drug use				0.51	0.48
Yes	382 (19.7)	9 (24.3)	373 (19.6)		
No	1555 (80.3)	28 (75.7)	1527 (80.4)		

In the preceding six months before the latest HIV test, 37.2% of participants reported two or more sexual partners, 4.5% reported having HIV-positive sex partners, while 48.3% did not know the HIV status of their sexual partners, and those who never or occasionally used condoms during anal sex accounted for 35.8%. The percentage of MSM engaging in receptive anal sex exclusively and MSM engaging in insertive anal sex exclusively were 13.1% and 44.1%, respectively. Among 51.7% who ever had heterosexual sex, 37.5% reported inconsistent condom use. A minority (8.2%) reported engaging in group sex, 3.9% reported being diagnosed with STIs, and 19.7% reported recreational drug use in the preceding six months.

### Univariate analysis of risk factors associated with HIV seroconversion (Table 2)

There were no statistically significant differences in sociodemographic characteristics between men with incident HIV and those who stayed HIV-negative (*P* > 0.05, **Table ****1**). Sexual behaviors in the past six months including higher number of sexual partners, having a HIV-positive partner, inconsistent condom use during anal sex, engaging in exclusively receptive or both insertive and receptive anal sex, inconsistent condom use during heterosexual sex, and being diagnosed with STIs were associated with incident HIV (*P* < 0.05). Having anal sex, participation in group sex, engaging in heterosexual sex, and recreational drugs use were not associated with HIV incidence (*P* > 0.05).

### Multivariate analysis of risk factors of HIV seroconversion (Table 3)

**Table 3.** Factors associated with HIV seroconversion identified using a proportional hazards regression model.

	HR (95% *CI*)	*P* value
Number of sexual partners		
0–1	–	–
2–5	1.30 (0.64–2.67)	0.47
≥ 6	2.65 (1.04–6.67)	0.04
Using condom for anal sex		
Never	–	–
Sometimes	0.72 (0.20–2.56)	0.61
Every time	0.27 (0.07–0.96)	0.04
HIV-positive partners		
No	–	–
Yes	3.82 (1.16–12.64)	0.03
Not sure	2.02 (0.95–4.30)	0.07
Sex role for anal sex		
Exclusively receptive	–	–
Exclusively insertive	0.23 (0.08–0.62)	< 0.01
Versatile	0.93 (0.46–1.88)	0.84

*HR* hazard ratio, *CI* confidence interval,–means not applicable

Proportional hazards regression model was conducted with statistically significant variables identified by univariate analysis (*P* < 0.10) as independent variables: occupation, the number of sexual partners, whether having HIV positive partners, frequency of condom use during anal sex, sex role during anal sex, frequency of condom use during heterosexual sex, frequency of condom use during heterosexual sex and diagnosed with STIs within the past six month before the latest HIV test. Having more than five sexual partners [hazard ratio (HR) = 2.65, 95% *CI*: 1.04–6.67], sex with partners living with HIV (HR = 3.82 95% *CI*: 1.16–12.64) were risk factors for HIV seroconversion. Consistent condom use for anal sex (HR = 0.27, 95% *CI*: 0.07–0.96), and being exclusively insertive for anal sex (HR = 0.23, 95% *CI*: 0.08–0.62) were protective factors for HIV seroconversion.

## Discussion

This is the largest online cohort study constructed and implemented through a GSN app globally that reports HIV incidence and its correlates among MSM. We add to current literature [13, 14, 39–44] by providing more robust estimates of HIV incidence, especially to the limited literature on app-using MSM [45]. Using this innovative methodology of GSN apps to maintain a cohort has advantages of reaching high-risk MSM and overcomes barriers related to fear of stigma and discrimination associated with offline cohort studies. Moreover, the app-assisted questionnaire may improve the data quality of sexual and highly stigmatized behaviors research among MSM. Data quality depends not only on the accurate recall of facts but also the degree of peoples’ self-disclosure, which is commonly influenced by an individual’s inherent need to create and maintain favorable impressions of oneself in the eyes of others. Therefore, MSM may misrepresent their true behaviors to avoid the stigma of homosexuality and the resulting discrimination in research with traditional methods. Studies reported that increased self-disclosure of sensitive information were found with decreased personal interactions with an interviewer [46, 47]. We conducted our research using a popular GSN app that was trusted by Chinese MSM; and surveys could be completed using the participants’ own smartphone, avoiding face-to-face interaction with research or healthcare staff. This strategy contributed to the acceptability of the study and consequently low drop-out rate.

Though studies have indicated that men using GSN apps could facilitate sexually risky behaviors and thus increase their HIV risk[18–23], the HIV incidence we calculated among app-using MSM was not higher than those recruited by traditional methods in 2012[14], or mixed method of respondent-driven sampling and internet in 2011[41] in Beijing. Given the differences in the establishment and follow-up methods employed in our cohort and others, it is not advisable to make any direct comparison of the incidence calculated in present study and others. Ongoing intervention efforts, changes in the patterns of HIV risk behaviors among subpopulations of MSM, difference in recruitment and following-up methods might all contribute to differences in reported HIV incidence. The factors associated with HIV seroconversion in our study were mostly consistent with other literatures using traditional methods. Having multiple sexual partners has been demonstrated to be a risk factor for acquiring HIV independent of condom use during sex [41, 42]. The number of sexual partners is also widely recognized as a predictor of the likelihood of acquiring STIs [48]. Studies revealed that GSN app-using MSM reported higher number of sex partners in China [19] and other countries [18, 24]. GSN smartphone app users could quickly and conveniently locate potential sex partners nearby, leading to more casual sex, greater number of sex partners, thus facilitate app users engaging in condomless anal sex [49, 50]. We found consistent condom use for anal sex reduced the probability of HIV incidence, which was inconsistent with other studies from MSM in Beijing with smaller sample sizes (348 [41], 574–769 [42]). This inconsistency might be due to their smaller sample sizes restricting the studies’ power to detect the independent effect of condoms use [41, 42].

About half of our participants were not aware of the HIV status of their partners; sex with partners living with HIV was a significant predictor of HIV incidence in our study, though only 4.5% reported having partners living with HIV. Previous research demonstrated that GSN smartphone app users were more likely to have a greater number of sexual partners known to have HIV and other STIs [23]; this increased their risk for HIV and STIs acquisition or transmission, compared with MSM who sought sex partners using other ways [26, 27]. Though seroadaptive practices (choosing HIV-negative partners or practicing only insertive anal sex with potentially discordant partners) among HIV-negative MSM was associated with a lower HIV incidence [51], the low disclosure of a partners’ HIV status increased HIV transmission among GSN smartphone app-using MSM. Further, among MSM in China, the lifetime HIV testing rates remained at only 47%, and the annual HIV testing rates was even lower (38%) [52], therefore 62% to 87% of MSM living with HIV remained undiagnosed [53, 54].

MSM exclusively practicing insertive anal sex had a reduced risk of HIV in our study, which was consistent with other studies [55–58]. In the United States, men who only had insertive anal sex had a lower risk of acquiring HIV (HR = 0.55, 95% *CI*: 0.36–0.84) [51]. Though some studies found sociodemographic characteristics like age and immigration status as correlates of HIV infection [42, 59–62], we did not find the same associations. Some reasons might be related to different sampling methods, different sample sizes and geographical difference [4, 63, 64].

Though China has scaled up its responses to HIV pandemic [65], our findings suggest that HIV incidence among GSN apps using MSM in Beijing was still high. The possible reason might be the patterns of HIV risk behaviors among MSM are changing with the use of the internet and GSN smartphone apps as the main tool for partner seeking, while efforts to control HIV transmission in this population still focused on individual-level behaviors, such as consistent condom use and regular HIV testing. Many studies demonstrated the importance of contextual factors—such as where MSM meet their partners—might have on the risk of HIV acquisition [18, 32, 45, 66]**.** GSN smartphone apps could facilitate partner seeking, thus result in more casual and condomless sex. Moreover, with the shift of partner seeking behaviors from in person venues to GSN apps, traditional intervention strategies designed for gay-centered venues, such as gay bars and public bathrooms, may not be effective in reaching MSM anymore. Our findings suggest that further strategies and implementation of new interventions are urgently needed to curb the HIV epidemic among MSM in China.

The main strength of our study is in its innovative use of GSN app to construct and maintain a cohort of MSM in China. Though there are studies among MSM recruited using social networking applications in China, most of them are cross-sectional studies [67, 68]. To our best knowledge, this study is the first cohort study constructed and implemented through a GSN app globally. With fear of stigma, MSM prefer to access information about HIV and testing services through the internet because the process is convenient, anonymous and private [69, 70]. With the development of network technology and improved internet access, more MSM seek health services, especially HIV-related services, through the internet [71, 72]; there is great potential for future use of GSN app in HIV research and intervention. In addition, we constructed an open cohort to estimate HIV incidence among app-using MSM, participants could complete a follow-up whenever they visited the GSN app for an HIV test, and new participants were enrolled whenever they visited the app to have an HIV test. Therefore, we obtained a cohort with participants staying natural and with better representativeness. Moreover, our cohort is still open for enrollment and follow-up; thus more MSM will be included and more data collected, to improve the robustness of future analyses.

Our study is subject to several limitations. First, our study population were mostly young men who used social network application for partner seeking and health services, so our findings are not generalizable to MSM who do not use social network applications. Thus, our estimated incidence may not be comparable with other reports of incidence from cohorts of MSM not using social network applications. MSM who use social network applications may be more likely to be more knowledgeable about HIV through educational campaigns on platforms like Blued, and so HIV incidence may be lower compared to other MSM. On the other hand, users of social network applications may have a higher risk for HIV as they may be more likely to have more sexual partners [18–23], and thus HIV incidence may be higher compared to other MSM. Second, some participants might test for HIV in other places but not in Blued sites during the study period. For example, they could directly go to the CDC to get an HIV test, therefore the HIV incidence we calculated may be underestimated.

Though China has scaled up its responses to the HIV epidemic [65], the HIV incidence among MSM has not significantly decreased. Given the pervasive use of GSN smartphone app among MSM and the changed patterns of sexual risk behaviors, further strategies and implementation of new interventions are urgently needed to curb the HIV epidemic among MSM in China. Our study demonstrated the strength of GSN smartphone app in conducting research among MSM, indicating that tailored interventions based on GSN smartphone app using can be more targeted and individualized, therefore may provide us with novel opportunities to decrease HIV infection and transmission of MSM.

## Conclusions

HIV incidence among GSN smartphone app-using MSM in Beijing was high. Interventions tailored to this population should strengthened. As a new tool for accessing MSM at higher risk for HIV/STIs transmission, GSN smartphone app could potentially play an important role in HIV control among MSM.

## Data Availability

The datasets upon which our findings are based belong to Blued and Nankai University, and the study is still ongoing. For confidentiality reasons, the datasets are not publicly available. However, the datasets can be availed upon reasonable request from the corresponding author and with permission from Blued and Nankai University.

## Abbreviations

MSM: Mem who have sex with men
HIV: Human immunodeficiency virus
AIDS: Acquired immunodeficiency syndrome
GSN: Geosocial networking
App: Application
CDC: Center for Disease Control and Prevention
STIs: Sexually transmitted infections
UID: User identification number
